# *Bacillus amyloliquefaciens* SAY09 Increases Cadmium Resistance in Plants by Activation of Auxin-Mediated Signaling Pathways

**DOI:** 10.3390/genes8070173

**Published:** 2017-06-28

**Authors:** Cheng Zhou, Lin Zhu, Zhongyou Ma, Jianfei Wang

**Affiliations:** 1 Key Laboratory of Bio-Organic Fertilizer Creation, Ministry of Agriculture, Anhui Science and Technology University, Bengbu 233100, China; czhou1224@hotmail.com; 2 School of Life Science and Technology, Tongji University, Shanghai 200092, China; putaojiuvsduyao@126.com

**Keywords:** volatile organic compounds, iron uptake, auxin, cadmium stress, nitric oxide

## Abstract

Without physical contact with plants, certain plant growth-promoting rhizobacteria (PGPR) can release volatile organic compounds (VOCs) to regulate nutrient acquisition and induce systemic immunity in plants. However, whether the PGPR-emitted VOCs can induce cadmium (Cd) tolerance of plants and the underlying mechanisms remain elusive. In this study, we probed the effects of *Bacillus amyloliquefaciens* (strain SAY09)-emitted VOCs on the growth of *Arabidopsis* plants under Cd stress. SAY09 exposure alleviates Cd toxicity in plants with increased auxin biosynthesis. RNA-Seq analyses revealed that SAY09 exposure provoked iron (Fe) uptake- and cell wall-associated pathways in the Cd-treated plants. However, SAY09 exposure failed to increase Cd resistance of plants after treatment with 1-naphthylphthalamic acid (NPA) or 2-(4-carboxyphenyl)-4,4,5,5-tetramethylimidazoline-1-oxyl-3-oxide (c-PTIO). Under Cd stress, SAY09 exposure markedly promoted Fe absorption in plants with the increased hemicellulose 1 (HC1) content and Cd deposition in root cell wall, whereas these effects were almost abrogated by treatment with NPA or c-PTIO. Moreover, exogenous NPA remarkably repressed the accumulation of nitric oxide (NO) in the SAY09-exposed roots under Cd stress. Taken together, the findings indicated that NO acted as downstream signals of SAY09-induced auxin to regulate Fe acquisition and augment Cd fixation in roots, thereby ameliorating Cd toxicity.

## 1. Introduction

Soils contaminated by heavy metals have become one of the most serious problems because of the dispersal of industry wastes, over-use of phosphate fertilizers, and atmospheric deposition [1]. Cadmium (Cd) is one of common heavy metals, which adversely affect plant growth, productivity and quality worldwide [2]. Cd is water-soluble and deposits in the surface layer of soils, which can be readily absorbed by plant roots and transferred into aboveground tissues [3]. Cd easily enters into the human food chain and poses an increasingly severe threat to public health [4]. Thus, there is an urgent need to develop sustainable strategies for remedying Cd-contaminated soils and reducing Cd accumulation in plants.

During long-term evolution, plants have developed diverse strategies to detoxify Cd, such as cell wall binding, chelation with phytochelations (PCs), and regulation of Cd distribution in plants [3]. The ability of plants to tolerate Cd stress varies with different plant species, but most plants often display leaf chlorosis, stunned growth, and inhibition of photosynthesis under Cd stress [2,5]. Cd is chemically similar to some essential elements for plant growth, including calcium (Ca), zinc (Zn) and iron (Fe), and it can enter into plant cells by Ca, Zn and Fe transporters/channels [3]. Intriguingly, Cd exposure triggers Fe deficiency responses in plants such as barley [6], *Arabidopsis* [7], and tobacco [8]. It has previously been indicated that Cd toxicity is primarily ascribed to its competition with other essential elements, particularly Fe, for metal-binding molecules [9]. Many studies have indicated that *IRT1*, encoding a major Fe transporter of the strategy I plants, can transport Cd from the soils into plant roots [10,11]. Exogenous gibberellic acid (GA) [12] or abscisic acid (ABA) [13] ameliorates Cd toxicity in *Arabidopsis* plants by downregulating the transcription of *IRT1*. Furthermore, nitric oxide (NO) is an important gaseous molecule that plays an important role in alleviating Cd toxicity in plants [14,15,16]. NO treatment has been shown to increase Cd resistance in rice by enhancing Cd retention in roots [17]. Similarly, auxin applied exogenously can markedly enhance the ability of *Arabidopsis* plants to tolerate Cd stress by increasing the Cd fixation in root cell wall [18]. 

Recently, microbial community habiting in the plant rhizosphere has attracted considerable attention. Plants release carbon compounds into the rhizosphere for increasing microbial activity and biomass [19]. Some beneficial free-living bacteria are collectively referred to as plant growth-promoting rhizobacteria (PGPR), which facilitate plants counteracting soil-borne plant pathogens, and regulate plant growth and development by a wide ranging of mechanisms, such as the production of phytohormones, siderophores, volatile organic compounds (VOCs), and protection enzyme [20,21,22]. Thus, a large number of studies have reported that PGPR can improve the ability of plants to tolerate abiotic stress, such as drought, high salinity, and nutrient deficiency [23,24,25,26,27]. Importantly, PGPR have also been shown to increase the resistance of host plants to Cd stress [28,29,30]. High Cd-resistant soil bacteria can alleviate Cd toxicity and reduce Cd absorption in pumpkin and mustard plants by increasing production of siderophore [28]. More recently, Cd-tolerant PGPR strains remarkably reduce Cd absorption and its accumulation of rice grains [29,30]. Thus, PGPR have the great potential for reducing plant uptake of Cd and inhibiting its translocation from roots to aboveground tissues. 

Until now, PGPR have been demonstrated to regulate physiological processes of host plants without physically contacting with plant roots by releasing VOCs [31,32,33,34]. Recently, the PGPR-emitted VOCs have been taken as novel regulators during plant-microbe interaction [35]. Microbial VOCs do not contain any hormones such as ABA, GA and indole-3-acetic acid (IAA), but endogenous hormone levels in host plants can be mediated by microbial VOCs, implying that the production of VOCs by PGPR acts as important messengers for the microbe-plant communication, and further regulates plant growth and development [36,37]. *Bacillus subtilis* GB03 VOCs induces auxin biosynthesis and regulates cell expansion in *Arabidopsis* plants [36]. The emission of VOCs by GB03 sufficiently activates the transcription of *FIT1, IRT1,* and *FRO2* in plants, thereby promoting Fe absorption [37]. Thus, a wide range of bacterial species such as *Paenibacillus* [38], *Bacillus* [36], and *Pseudomonas* [39] can release diverse kinds of VOCs to regulate nutrient uptake, abiotic and biotic stress responses in plants. Nevertheless, whether microbial VOCs could improve plant’s adaption to Cd stress and the underlying mechanisms are still unclear. 

In the present study, a bacterial strain that was highly resistant to 1.5 mM CdCl_2_ was isolated and identified as *Bacillus amyloliquefaciens* (strain SAY09). The release of VOCs by SAY09 conferred increased Cd tolerance in *Arabidopsis* plants. We further addressed the question how SAY09 VOCs ameliorated Cd toxicity in plants. Biochemical, transcriptomic, and pharmacological analyses were combined to unravel the mechanisms of SAY09-induced Cd tolerance of plants. The results demonstrated that SAY09 exposure ameliorated Cd toxicity in plants by induction of auxin accumulation, thereby enhancing Fe acquisition and Cd retention in root cell wall.

## 2. Materials and Methods 

### 2.1. Isolation of Bacteria Strain and Identification of VOCs

For the isolation of Cd-tolerant bacteria, about 1.0 g Cd-contaminated soil was cultured in 10 mL of sterile water with shaking at 180 rpm for 1 h at 28 °C. Then, 1 mL of the suspension was serially diluted from 10^−1^ to 10^−6^, and each diluted solution was spread on Luria Broth (LB) plates (10 g L^−1^ tryptone, 5 g L^−1^ yeast extract, 10 g L^−1^ NaCl, and 15 g L^−1^ agar) in the presence of 0.1–1.5 mM CdCl_2_, and incubated at 28 °C for 20 h. The growing colonies were inoculated on LB plates containing different Cd concentrations. Finally, the isolates of Cd-tolerant bacteria grown on the Cd-containing LB plates were selected for further analyses. One of bacteria strains was isolated and identified as *B. amyloliquefaciens* SAY09 by 16S rDNA sequencing (Genebank No. MF037705). 

This bacteria strain was cultured in liquid LB at 28 °C for 18 h, with shaking at 200 rpm. Subsequently, the cultured bacteria were diluted to an OD600 absorbance of 1.5 (10^7^ CFU mL^−1^) in sterile water. An aliquot of 30 μL of bacterial culture was used to expose plants to VOCs according to the method described by Asari et al. [40]. Moreover, the production of VOCs by SAY09 was identified using gas chromatography (GC)–mass spectrometry (MS) analyses as reported recently by Wang et al. [34]. 

### 2.2. Plant Materials and Growth Conditions

Seeds of *Arabidopsis thaliana* (ecotype Columbia) were surface sterilized with 0.1% HgCl_2_, and then rinsed at least three times with sterile water. The seeds were vernalized at 4 °C for 48 h and cultured on 1/2 Murashige and Skoog (MS) medium with 1.5% sucrose and 0.7% agar (pH 5.8). After 6 days (d) of germination, *Arabidopsis* seedlings were transferred to one side of petri dishes containing 1/2 MS agar medium, and the other side was inoculated with 30 μL of bacterial culture with or without the presence of 50 μM CdCl_2_, and were co-cultured for 12 d. The Petri dishes were placed on a growth chamber at 23 °C, 16/8 h (light/dark) photoperiod with 130 µmol m^−2^ s^−1^ light intensity.

### 2.3. Measurement of Fe and Cd Content

For assays of Cd treatment, 50 μM CdCl_2_ with or without 0.05 μM IAA or 50 μM S-nitrosoglutathione (GSNO) was added into 1/2 MS agar medium. After 12 d of treatment, shoots and roots of seedlings were separated and harvested, and then the samples were digested with HNO_3_/HClO_4_ (4:1, *v*/*v*) in a microwave system according to the method described by Lei et al. [41]. The content of Fe and Cd was measured by inductively coupled plasma atomic emission spectroscopy (ICP-AES; Perkin Elmer Optimal 2100DV, Norwalk, NY, USA). The content of cell wall-retained or soluble Fe was measured as reported by Lei et al. [41]. In addition, cell wall-retained Cd was extracted by 2 N HCl for 24 h, and then the content of Cd was determined using ICP-AES. 

### 2.4. Measurement of Photosynthetic Parameters

To measure total chlorophyll content in leaves, 100 mg of rosette leaves were extracted with 1.5 mL of ethanol (95%, *v*/*v*) at room temperature in dark place. Absorbance of the extracted chlorophyll was recorded at wavelengths of 649 and 665 nm. The chlorophyll levels were calculated as follow: A665 × 13.95–A649 × 6.88 as reported by Zhao et al. [42]. In addition, net photosynthetic rate (Pn) of *Arabidopsis* leaves was determined using an open-flow gas-exchange system (LI-6400; LI-COR, Lincoln, NE, USA). The fluorescence parameters including Fv/Fm and ΦPSII and were analyzed on rosette leaves after 1 h of dark adaptation using a chlorophyll fluorescence imaging systems FluorCam 7 (Photon Systems Instruments, Brno, Czech Republic). In each experiment, at least six leaves were tested, and three independent experiments were performed.

### 2.5. Analyses of Cellular ROS, Electrolyte leakage (EL) and Malondialdehyde (MDA) Levels

The content of reactive oxygen species (ROS) including H_2_O_2_ and O_2_^−^ was measured using 5-(and 6)-carboxy-2,7-dichlorodihydrofluorescein diacetate (H_2_DCFDA) and dihydroethidium (DHE), respectively. About 500 mg of tissue samples were homogenized with 2 mL of 50 mM Tris-HCl buffer (pH 7.0) followed by centrifugation at 12,000 × g for 10 min at 4 °C. Then, 200 µL of the supernatant was added into 1.8 mL of a reaction solution containing 50 mM Tris-HCl buffer, 10 µM H_2_DCFDA or DHE at 37 °C. After 30 min of dark incubation, the reaction solution was used to assay the content of H_2_O_2_ and O_2_^−^ using a FACScan flow cytometer (Becton Dickinson, Mountain View, CA, USA) as reported by Chen et al. [43].

Electrolyte leakage (EL) was assayed according to the method reported by Huo et al. [44] with minor modification. Leaf discs (5 mm in diameter) were placed in 10 mL of distilled water and then vacuumed for 10 min, followed by incubating at room temperature for 10 h. Initial electrical conductance (C1) was determine using a DDB-303A conductivity meter (Ningbo Biocotek Scientific Instrument, Ningbo, China). Subsequently, the mixture was boiled for 30 min and then cooled to room temperature for measuring the final electric conductance (C2). Finally, EL was calculated according to the formulae: EL (%) = C1/C2 × 100. In addition, the malondialdehyde (MDA) content in leaves was measured based on the thiobarbituric acid (TBA)-based colorimetric method as described by Draper et al. [45].

### 2.6. RNA-Sequencing (RNA-Seq) Analyses

Total RNA was extracted from the non-exposed (control) and SAY09-exposed plants grown under non-treated or Cd stress conditions using Trizol reagent (Invitrogen, Carlsbad, CA, USA), the contaminated DNA was digested by DNase (Invitrogen). Then, the RNA quality and quantity was assayed by the Agilent 2100 Bioanalyzer (Agilent, Santa Clara, CA, USA). In addition, 500 ng of total RNA from three independent plants in each experimental group was pooled to construct four cDNA libraries. The average length of the cDNA fragments was about 250 bp. The 15 bar-coded cDNA libraries were pooled, and single-end sequencing was conducted using the Hiseq 2500 platform (Illumina, San Diego, CA, USA). The raw data were processed by removing the adaptor sequences and low-quality reads, and were then deposited into the National Center for Biotechnology Information (NCBI) Sequence Read Archive (SRA) database (accession No. SRR5487323). Gene ontology (GO) analysis was performed using the Blast2GO program. Differentially expressed genes (DEGs) were identified using the analysis package DEGseq at FDR-adjusted *p*-value < 0.05, and were assigned to GO term [34]. 

### 2.7. Assays of ABA, IAA and GA Content, and Glucuronidase (GUS) Staining

The content of ABA, IAA and GA was measured by high performance liquid chromatography (HPLC) coupled with mass spectrometry (MS) according to the method described by Sofo et al. [46]. For GUS staining, the roots of DR5::GUS *Arabidopsis* seedling were immersed in aqueous acetone (90%, *v*/*v*) for 20 min, followed by transferring into the staining solution containing 0.2% (*v*/*v*) Triton X-100, 2 mM potassium ferrocyanide, 1 mM 5-bromo-4-chloro-3-indolylglucuronide (X-Gluc), 2 mM potassium ferricyanide for 12 h at 37 °C. The samples were photographed using a Nikon Eclipse 80i microscope (Tokyo, Japan).

### 2.8. Detection of NO Content in Roots

The NO accumulation in roots was analyzed using the 4-amino-5-methylamino-2′,7′-difluorofluorescein diacetate (DAF-FM DA). About 2 cm segments from root apices were separated and immediately incubated in the 2-[4-(2-hydroxyethyl)-1-piperazinyl]ethanesulfonic acid (HEPES)-NaOH solution containing 2 μM DAF-FM DA (pH 7.5) for 30 min in the dark. Then, the root samples were rinsed three times with HEPES-NaOH solution. The NO-associated fluorescence was imaged by a Leica SP2-AOBS confocal microscope (Leica, Wetzlar, Germany) with an excitation filter of 488 nm and an emission filter of 515 nm, respectively. 

### 2.9. Analyses of Cell Wall Extraction and Compositions

Based on the method described by Zhu et al. [47], crude cell wall was prepared and fractionated into different kinds of fractions such as hemicellulose 1 (HC1) and pectin. Total polysaccharide content in HC1 was measured as reported by Dubois et al. [48]. Briefly, the extracted HC1 were treated with H_2_SO_4_ and phenol at 23 °C for 15 min followed by 100 °C for 15 min. Then, the absorbance was recorded spectrophotometrically at 490 nm. In addition, the uronic acid content in pectin was determined using galacturonic acid as a standard as reported by Zhu et al. [47]. The extracted HC1 were subjected to a series of treatments with H_2_SO_4_, Na_2_B_4_O_7_, and M-hydro-dipheny. The absorbance was recorded spectrophotometrically at 520 nm. 

### 2.10. qRT-PCR Analyses

Total RNA was extracted from roots using Trizol reagent (Invitrogen) following the manufacturer’s instructions. DNA contamination in RNA samples was digested by DNase (Invitrogen). About 500 ng of total RNA was reversely transcribed into first-strand cDNA using the PrimeScript RT reagent kit (Takara, Tokyo, Japan). Quantitative real time PCR (qRT-PCR) analysis was carried out in an Applied Biosystems (ABI) 7500 PCR machine (Applied Biosystems, Carlsbad, CA, USA) using the following reaction conditions: 30 s at 94 °C, 30 s at 95 °C, 30 s at 60 °C, 30 s at 72 °C for 40 cycles. The *Arabidopsis* actin2 was used as an internal reference for normalizing target gene expression. The pairs of gene-specific primers used in this study were listed in Table S1. 

### 2.11. Ultrastructural Observation and Cd Localization by Transmission Electron Microscopy Analyses

Leaf samples were cut into small pieces of about 0.5 cm^2^, and immediately fixed with 2.0 % glutaraldehyde in 0.1 M phosphate buffer saline (PBS, pH 7.2) for 6 h followed by three rinses with PBS. Then, the samples were fixed with 2.0 % OsO_4_ for 1 h. After three rinses, the samples were dehydrated and embedded in Spurr’s resin (Ted Pella, Redding, CA, USA). The embedded materials were cut into thin sections (100 nm) and observed by a JEM-1230 transmission electron microscope (JEOL; Tokyo, Japan) at 80 kV. Furthermore, for analyzing Cd localization, leaf samples were fixed in Na_2_S (1%, *m*/*v*) at room temperature for 1 h. Then, the samples were subjected to a series of treatments such as dehydration and embedding, and were lastly cut into thin sections (100 nm) for TEM analyses as described previously by Wójcik and Tukiendorf [49]. 

### 2.12. Statistical Analyses

Each experiment was carried out at least three biological repeats. The data were analyzed by one-way analysis of variance (ANOVA) using Duncan’s multiple range test. The bars represented the mean and standard deviation (SD). Different letters above the histograms indicated that the means were significantly different among different groups at *p* < 0.05. 

## 3. Results

### 3.1. SAY09 Exposure Increases the Tolerance of Arabidopsis Plants to Cd Stress

**Figure 1:**
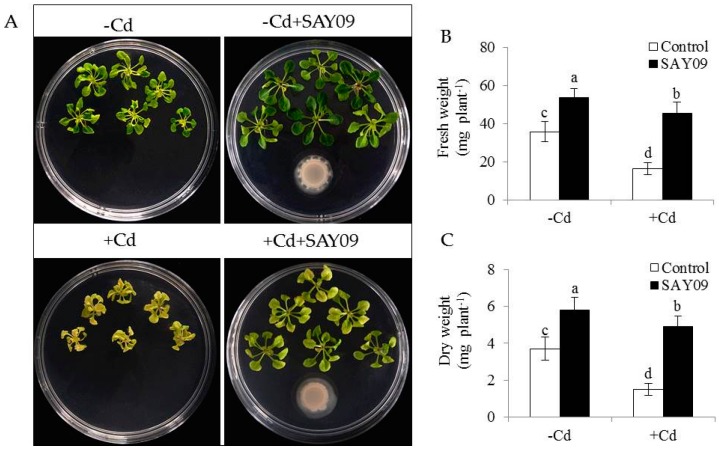
*B. amyloliquefaciens* SAY09 increases the resistance of *Arabidopsis* plants to Cd stress. Six-day-old seedlings were cultured on 1/2 Murashige and Skoog (MS) agar medium with or without 50 μM CdCl_2_, after 12 d of exposure to SAY09 volatile organic compounds (VOCs), and non-exposed plants as the controls. Then, these plants were used to examine the growth phenotypes (**A**), fresh weight (**B**), and dry weight (**C**). Different letters above each bar indicate significant difference at *p* < 0.05.

**Figure 2:**
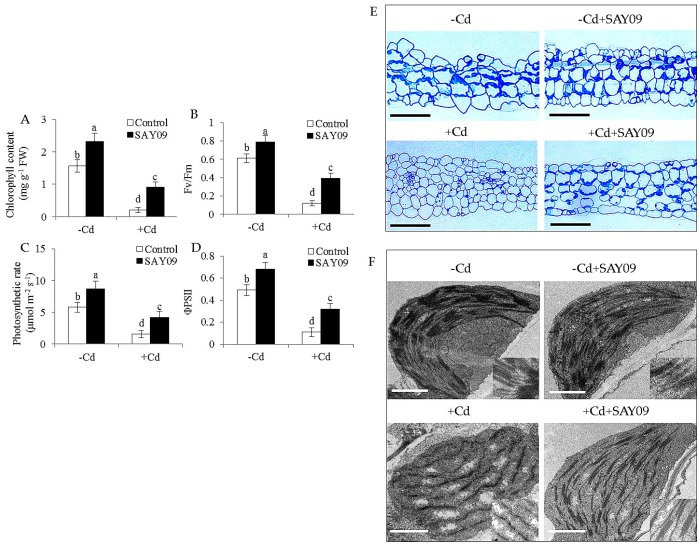
Effects of SAY09 exposure on the photosynthesis and chloroplast development under Cd stress. Six-day-old seedlings were cultured on 1/2 MS agar medium with or without 50 μM CdCl_2_, after 12 d of exposure to SAY09 VOCs, and non-exposed plants as the controls. Then, these plants were used to measure total chlorophyll content (**A**), maximum quantum yield of photosystem II (PSII (Fv/Fm) (**B**), photosynthetic rate (**C**), ΦPSII (**D**), leaf sections; Scale bar = 30 μm. (**E**) and chloroplast ultrastructure; Scale bar = 1 μm. (**F**). Different letters above each bar indicate significant difference at *p* < 0.05.

To investigate whether SAY09-emitted VOCs improved the resistance to Cd toxicity in *Arabidopsis* plants, the growth traits of both the control and SAY09-exposed plants were examined. Initially, the composition of VOCs released by SAY09 was extracted and detected by GC–MS. In addition, 19 different kinds of volatile compounds were identified (Table S2). Under non-stress conditions, after 12 d of exposure to SAY09 VOCs, plants exhibited better growth performance with increased fresh and dry weight compared with the controls (Figure 1A–C), indicating a promoting role of SAY09 VOCs in plant growth. On the media containing 50 μM CdCl_2_, the controls displayed chlorotic leaves with reduction of fresh and dry weight. In contrast, the SAY09-exposed plants exhibited greener leaves and higher biomass under Cd stress (Figure 1A–C). Total chlorophyll content was greatly higher in the SAY09-exposed plants than that in the controls under non-stress and Cd stress conditions (Figure 2A). Additionally, the SAY09-exposed plants displayed higher photosynthetic efficiency with the increased the values of maximum quantum yield of photosystem II (PSII) (Fv/Fm), photosynthetic rate (Pn), and ΦPSII compared with the controls (Figure 2B–D). 

**Figure 3:**
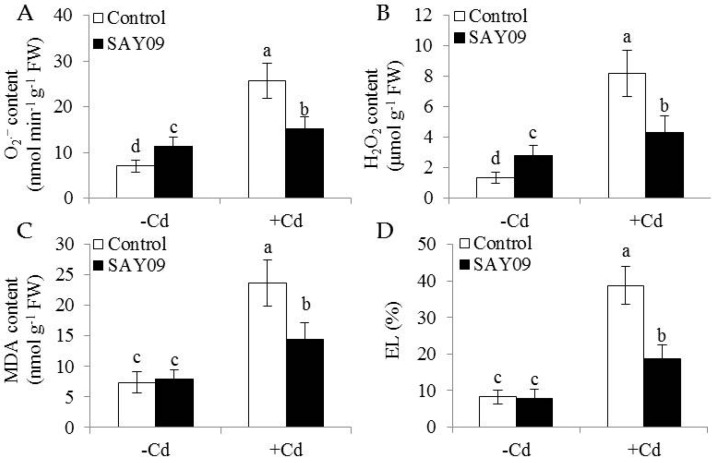
Effects of SAY09 exposure on the levels of ROS, malondialdehyde (MDA) and Electrolyte leakage (EL) in *Arabidopsis* plants grown under Cd stress. Six-day-old seedlings were cultured on 1/2 MS agar medium with or without 50 μM CdCl_2_, after 12 d of exposure to SAY09 VOCs, and non-exposed plants as the controls. Then, these plants were used to measure the levels of O_2_^−^ (**A**), H_2_O_2_ (**B**), MDA (**C**), and EL (**D**). Different letters above each bar indicate significant difference at *p* < 0.05.

Microscopic observation of leaf sections displayed a marked increase of chloroplast numbers in the SAY09-exposed plants under Cd stress compared with the controls, but no significant difference was observed under non-stress conditions (Figure 2E). Moreover, transmission electron microscopy (TEM) analyses of mesophyll cells showed that the chloroplasts of SAY09-exposed plants had greater granum lamellae than that of the controls under non-stress conditions (Figure 2F). After 12 d of Cd treatment, fully swollen chloroplasts and less granum lamellae occurred in plastids of mesophyll cells from the controls, whereas the chloroplasts of SAY09-exposed plants had more normal granum stacking than that of the controls. As shown in Figure 3, under non-stress conditions, a slight increase of the content of ROS including H_2_O_2_ and O_2_^−^ in the SAY09-exposed plants compared with the controls, whereas no significant difference in MDA and EL values was observed between the control and SAY09-exposed plants. Moreover, cellular ROS levels were remarkably increased in plants as consequence of Cd treatment, although the SAY09-exposed plants displayed lower ROS accumulation than the controls. A similarly changing tendency was observed for the values of MDA and EL.

### 3.2. SAY09 Exposure Increases Fe Acquisition with Reduced Shoot Cd Accumulation

**Figure 4:**
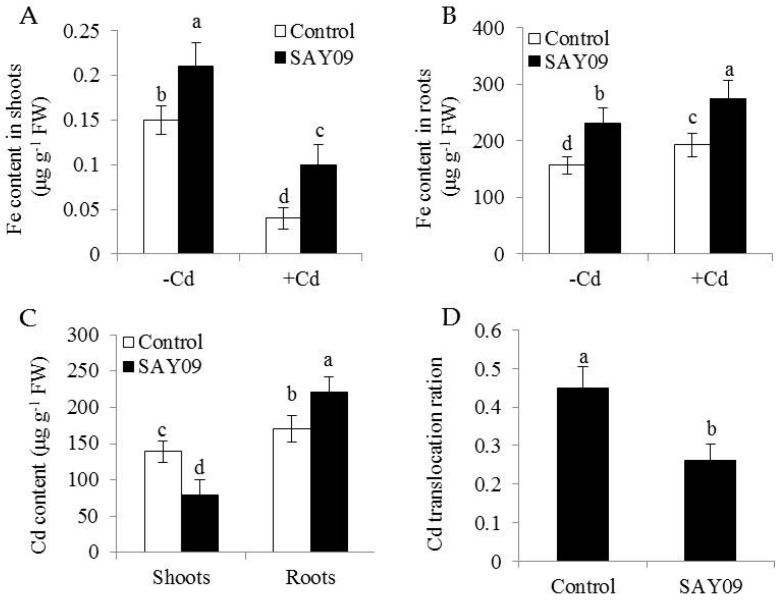
Effects of SAY09 exposure on the accumulation of Fe and Cd in *Arabidopsis* plants grown under Cd stress. Six-day-old seedlings were cultured on 1/2 MS agar medium with or without 50 μM CdCl_2_, after 12 d of exposure to SAY09 VOCs, and non-exposed plants as the controls. Then, these plants were used to measure shoot (**A**) and root (**B**) Fe content. In addition, the content of Cd (**C**) and its translocation ration (**D**) were further examined in both the control and SAY09-exposed plants grown under Cd stress. Different letters above each bar indicate significant difference at *p* < 0.05.

To examine if the SAY09-induced Cd tolerance of plants was tightly associated with the nutritional status of Fe, shoot and root Fe content was determined using ICP-AES. In shoots, the SAY09-exposed plants showed about 38% and 62% higher Fe content than the controls under non-stress and Cd stress conditions, respectively (Figure 4A). The content of Fe was about 32% and 28% higher in the SAY09-exposed roots, respectively (Figure 4B). 

**Figure 5:**
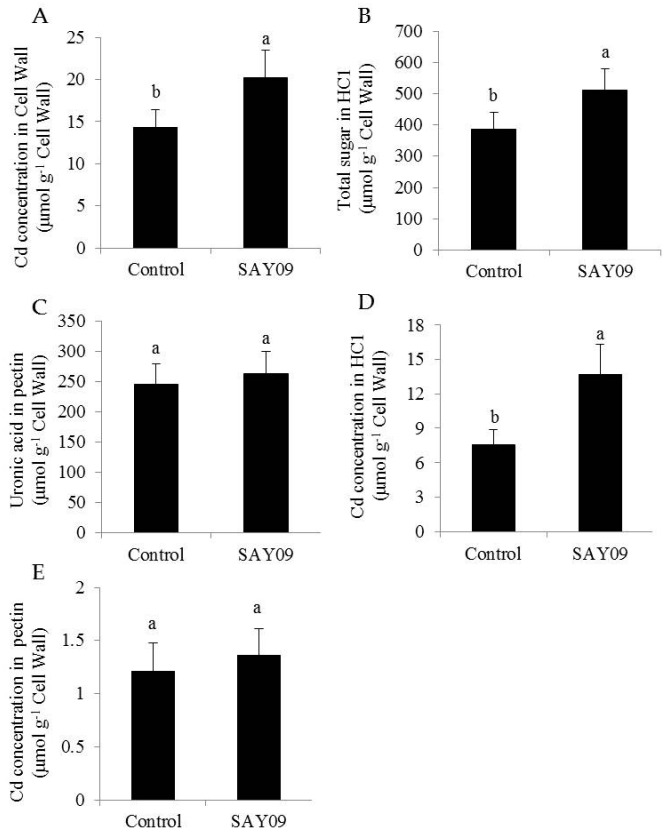
SAY09 exposure affects the Cd accumulation in *Arabidopsis* under Cd stress. Six-day-old seedlings were cultured on 1/2 MS agar medium with 50 μM CdCl_2_, after 12 d of exposure to SAY09 VOCs, and non-exposed plants as the controls. Then, these plants were used to measure Cd concentration (**A**), HC1 (**B**), and pectin (**C**) in root cell wall, and Cd concentration in HC1 (**D**) and pectin (**E**). Different letters above each bar indicate significant difference at *p* < 0.05.

Furthermore, the SAY09-exposed roots accumulated 22% higher Cd content than the controls after 12 d of Cd treatment (Figure 4C). However, the SAY09-exposed shoots had 39% lower Cd content than the controls. The ratio of Cd translocation from roots to shoots (shoot Cd amount/total Cd amount) was also examined. The SAY09-exposed plants showed a pronouncedly lower Cd translocation ratio than the controls (Figure 4D), implying that the SAY09-emitted VOCs possibly enhanced Cd fixation in roots and further reduced its translocation from roots to shoots. Thus, we investigated the Cd accumulation in the cell wall of both the control and SAY09-exposed plants. As shown in Figure 5A, the emission of VOCs by SAY09 induced a great increase of Cd accumulation in the cell wall of SAY09-exposed roots compared with the controls. Moreover, SAY09 exposure markedly increased HC1 content under Cd stress compared with the controls, but not for the content of pectin (Figure 5B,C). The amount of Cd was observably higher in HC1 of the SAY09-exposed plants than those of the controls, whereas there was no marked difference in the amount of Cd in pectin (Figure 5D,E).

### 3.3. Transcriptomic Analyses of SAY09-Regulated Genes in the Cd-Treated Plants 

To unravel the mechanisms that SAY09-emitted VOCs induced Cd tolerance in Arabidopsis plants, whole genome expression profiles was performed using RNA-Seq analyses. After 2 d of Cd treatment, the *Arabidopsis* plants were selected to explore the Cd-responsive genes because 50% of the maximum Cd content was found in the aboveground tissues at this time point (Figure S1). The exponential increase of Cd indicated an intensely metabolic regulation in plants, thus accompanying by the induction of abundant gene expression by Cd exposure. We next compared analyses of transcriptome profiles among the non-treated (NT), Cd-treated (CT), and Cd-treated SAY09-exposed plants (CS) plants with an FDR-adjusted *p*-value < 0.05 as the threshold. A total of 34,802,106, 34,646,664 and 34,770,310 raw reads were generated in the NT, CT and CS libraries by 454 sequencing, respectively, and the raw reads were submitted into the NCBI SRA database (accession No. SRR5388903). After eliminating adapter sequence and low quality reads, 32,552,016 (93.53%), 32,684,448 (94.34%), and 33,009,931 (94.94%) clean reads were remained in the NT, CT and CS libraries, respectively. Moreover, 31,496,094 (90.50%), 31,648,110 (91.35%) and 31,943,604 (91.87%) clean reads were uniquely mapped in the NT, CT and CS libraries, respectively (Table S3).

**Figure 6:**
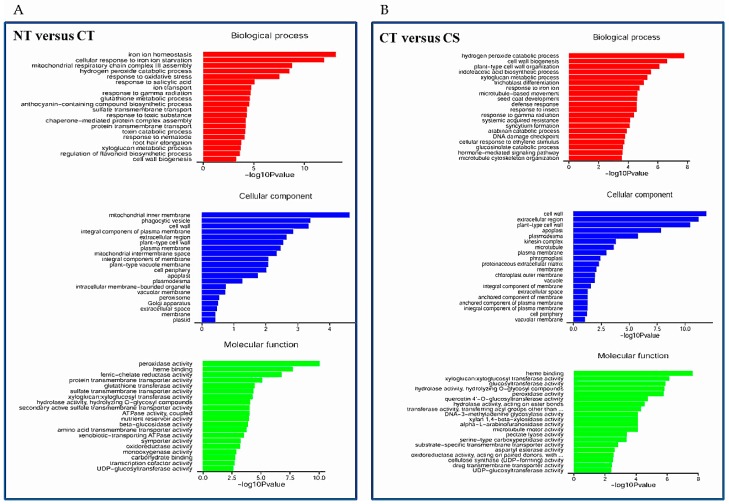
List of the predominant Gene Ontology (GO) terms for upregulated differentially expressed genes (DEGs) based on GO classifications. GO terms were categorized into three groups: biological process, cellular component and molecular function. (**A**) Non-treated (NT) plants and versus Cd-treated (CT) plants; (**B**) CT plants versus Cd-treated SAY09-exposed (CS) plants. *p*-value represents significant difference among NT, CT and CS libraries.

Compared with the NT library, there were 450 upregulated and 774 downregulated DEGs in the CT library (Table S4). Gene Ontology (GO) term annotations for the DEGs were clustered into three categories including molecular function, cellular component and biological process (Figure 6A). The predominant categories in the upregulated DEGs were mainly related to some important pathways such as ‘iron ion homeostasis’, ‘hydrogen peroxide catabolic process’, and ‘response to oxidative stress’ in biological processes; ‘mitochondrial inner membrane’, ‘vacuole membrane’ and ‘cell wall’ in the cellular component; ‘peroxidase activity’, ‘ferric-chelate reductase activity’ and ‘sulfate transmembrane transporter activity’ in molecular function. Among the downregulated DEGs, the predominant categories were involved in biological processes such as ‘photosynthesis’, ‘response to iron ion’, and ‘indoleacetic acid biosynthetic process’, followed by the molecular function and cellular process categories (Figure S2A). Furthermore, a total of 1055 genes including 459 downregulated and 596 upregulated genes were differentially expressed between the CT and CS libraries (Table S5). The most predominant categories in the upregulated DEGs was closely associated with several pathways such as ‘hydrogen peroxide catabolic process’, ‘cell wall organization’, ‘indoleacetic acid biosynthetic process’, and ‘xyloglucan metabolic process’ in biological processes; ‘cell wall’, ‘extracellular region’ and ‘apoplast’ in the cellular component; and ‘xyloglucan xyloglucosyl transferase activity’, ‘glucosyltransferase activity’ and ‘peroxidase activity’ in molecular function (Figure 6B). In addition, the predominant categories of biological processes in the downregulated DEGs were associated with ABA/stress-related pathways such as ‘response to water deprivation’, ‘response to cold’, and ‘response to abscisic acid’ (Figure S2B). Furthermore, qRT-PCR was used to verify gene transcriptional profiles that were observed in the DEGs (randomly selected eight genes: TCP1, NIT1, XTH12, CYP81D1, PDF2.1, PUP4, PRP1 and LTP2). The changing trend of gene expression was in accordance with that detected by RNA-Seq (Figure S3), indicating a high reliability of RNA-Seq data. 

### 3.4. SAY09 Exposure Positively Regulates Auxin Biosynthesis in Plants

**Figure 7:**
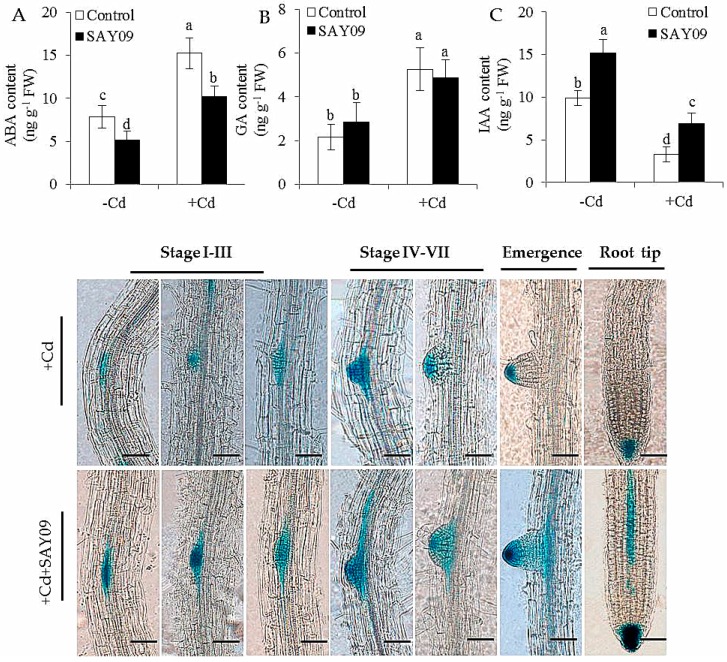
Accumulation of abscisic acid (ABA) (**A**), gibberellic acid (GA) (**B**) and indole-3-acetic acid (IAA) (**C**) in both the control and SAY09-exposed plants with or without 50 μM CdCl_2_. Moreover, the distribution of DR5::GUS was monitored in lateral roots and root tips under Cd stress after 48 h of SAY09 exposure (**D**) Scale bar = 0.3 mm.

In this study, the ABA content of the control and SAY09-exposed plants was measured. The SAY09-exposed plants had significantly lower ABA content than the controls (Figure 7A), indicating that the induction of Cd tolerance of plants by SAY09 was not attributable to activation of ABA-related signaling pathways. In addition, no significant difference in the content of GA was observed between the control and SAY09-exposed plants (Figure 7B). However, RNA-Seq analyses showed that SAY09 exposure drastically upregulated the expression of some genes involved in the biosynthetic process of IAA in the Cd-treated plants, but the transcription of these genes was distinctly downregulated in the controls grown under Cd stress (Table S6). 

With the time-delay of SAY09 exposure, the IAA content of SAY09-exposed roots exhibited a rapidly increasing tendency under non-stress conditions, but was not for the controls (Figure 7C). Moreover, the IAA levels were significantly decreased in the Cd-treated plants. However, the SAY09-exposed plants remained relatively high IAA content compared with the controls. Furthermore, the DR5::GUS *Arabidopsis* seedlings were used to examine the distribution of IAA in different stages of lateral root development under Cd treatments (Figure 7D). After 48 h of SAY09 exposure, strong GUS signals were observed during the formation of lateral roots in the Cd-treated plants, whereas only weak signals occurred in the entire stages of lateral root development in the controls. Moreover, the SAY09-exposed plants exhibited stronger GUS signals in root tips than the controls.

### 3.5. NPA Treatment Fails to Increase Cd Resistance in SAY09-Exposed Plants

**Figure 8:**
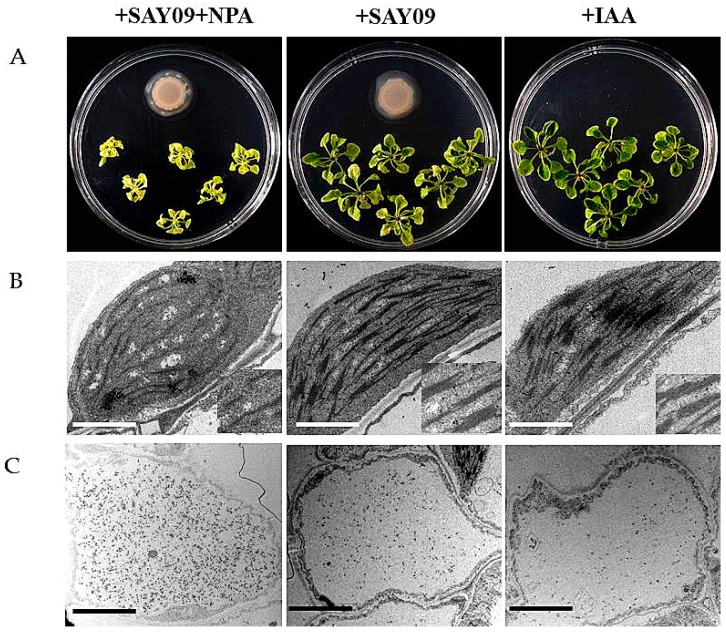
Effects of exogenous 1-naphthylphthalamic acid (NPA) and IAA on the Cd accumulation in *Arabidopsis* plants. Six-day-old seedlings were grown on 1/2 MS agar medium with 50 μM CdCl_2_ after 12 d of exposure to 2 μM NPA pulse SAY09 (+NPA+SAY09), SAY09 (+SAY09) or 0.05 μM IAA (+IAA) treatments. Then, these plants were used to analyze the phenotypes (**A**), chloroplast ultrastructure; Scale bar = 1 μm (**B**), and Cd deposition in the epidermis of leaves; Scale bar = 5 μm (**C**).

**Figure 9:**
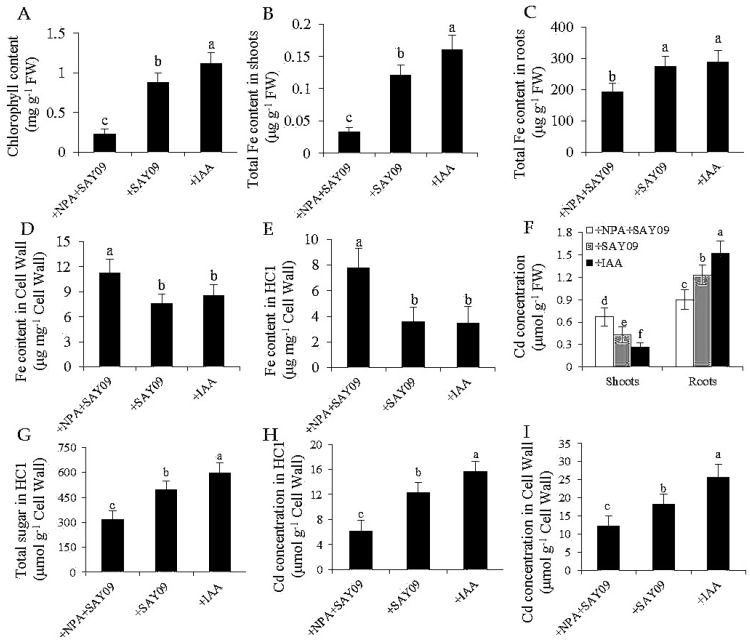
Effects of SAY09 exposure on total chlorophyll content (**A**), shoot (**B**) and root (**C**) Fe content, the Fe content in root cell wall (**D**) and HC1 (**E**), shoot and root Cd concentration (**F**), HC1 content (**G**), and the Cd concentration in root cell wall (**H**) and HC1 (**I**) in *Arabidopsis* plants grown under Cd stress. Six-day-old seedlings were grown on 1/2 MS agar medium with 50 μM CdCl_2_ after 12 d of exposure to 2 μM NPA pulse SAY09 (+NPA+SAY09), SAY09 (+SAY09) or 0.05 μM IAA (+IAA) treatments. Different letters above each bar indicate significant difference at *p* < 0.05.

To examine if auxin was required for SAY09-induced Cd tolerance of plants, 2 μM 1-naphthylphthalamic acid (NPA), an auxin transport inhibitor, was applied to treat the SAY09-exposed plants grown under Cd stress. As shown in Figure 8A, NPA treatment could not alleviate leaf chlorosis in plants under Cd stress after 12 d of SAY09 exposure. However, this chlorotic symptom was hardly observed in both the +SAY09 and +IAA treatments. Ultrastructural observation revealed that a relatively high amount of Cd deposited in the chloroplasts and epidermis of leaves in plants grown under +NPA+SAY09 treatment (Figure 8B,C). However, there was a lower amount of Cd deposited in leaves of plants grown under +SAY09 and +IAA treatments. Moreover, the mesophyll cells from Cd-exposed plants in +NPA+SAY09 treatment showed fewer photosynthetic lamellae in the plastids. By contrast, when the Cd-exposed plants were subjected to IAA and SAY09 treatments, the chloroplasts of mesophyll cells appeared fully developed and normal grana stacking and grana lamellae were prominently increased. Consistently, NPA treatment remarkably inhibited the SAY09-induced increase of chlorophyll content under Cd stress (Figure 9A).

Additionally, shoot and root Fe contents were much higher in both the +IAA and +SAY09 treatments relative to +NPA+SAY09 treatment, in agreement with the phenotype observed (Figure 9B,C). Furthermore, we measured the Fe content in the root cell wall. The results showed that the SAY09-exposed plants treated with NPA had significantly higher Fe content in HC1 and root cell wall than plants grown under +SAY09 and +IAA treatments, respectively, after 12 d of exposure to Cd stress (Figure 9D,E). We further determined the concentration of Cd in these plants. There were lower shoot Cd concentrations in plants grown under both +SAY09 and +IAA treatments versus +NPA+SAY09 treatment, although the SAY09- and IAA-exposed roots accumulated higher Cd concentration than those of plants grown under +NPA+SAY09 treatment (Figure 9F). Accordingly, the SAY09-emitted VOCs could not increase HC1 content in plants under Cd stress after NPA treatment, and thus did not augment Cd fixation in HC1 and the root cell wall (Figure 9G–I).

### 3.6. NO Acts Downstream of SAY09-Induced Auxin to Mediate Fe and Cd Absorption

**Figure 10:**
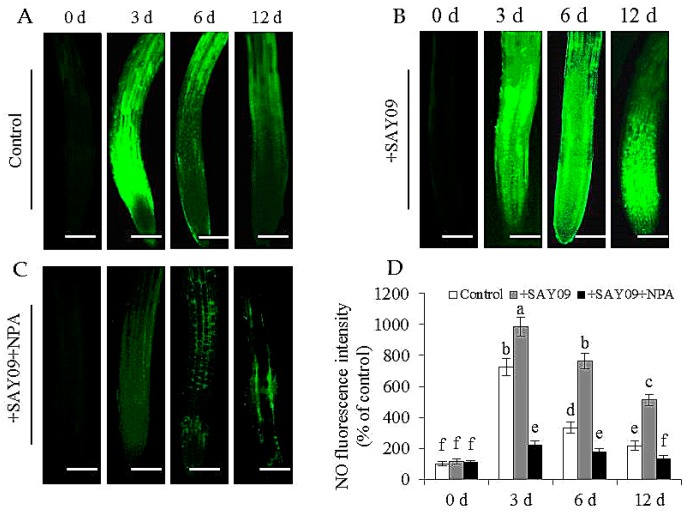
Effects of SAY09 exposure on the accumulation of NO in roots of non-exposed (control) (**A**), SAY09-exposed (+SAY09) (**B**), NPA-treated pulse SAY09-exposed (+NPA+SAY09) (**C**) *Arabidopsis* plants grown under Cd stress, and their corresponding relative fluorescence intensity in roots (**D**). Six-day-old seedlings were grown on 1/2 MS agar medium with 50 μM CdCl_2_ after 12 d of exposure to 2 μM NPA pulse SAY09 (+NPA+SAY09), SAY09 (+SAY09) or 0.05 μM IAA (+IAA) treatments. Scale bar = 1.0 mm Different letters above each bar indicate significant difference at *p* < 0.05.

**Figure 11:**
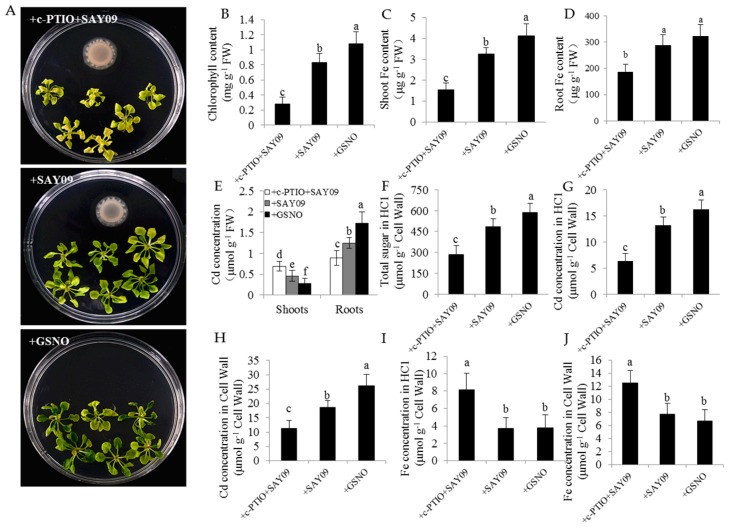
Effects of exogenous 2-(4-carboxyphenyl)-4,4,5,5-tetramethylimidazoline-1-oxyl-3-oxide (c-PTIO) on the Cd accumulation in *Arabidopsis* plants. Six-day-old seedlings were grown on 1/2 MS agar medium with 50 μM CdCl_2_ after 12 d of exposure to 1 mM c-PTIO pulse SAY09 (+c-PTIO+SAY09), SAY09 (+SAY09) or 50 μM GSNO (+GSNO) treatments. Then, these plants were used to analyze the phenotypes (**A**), total chlorophyll content (**B**), shoot (**C**) and root (**D**) Fe content, the Cd concentration in shoots and roots (**E**), HC1 content (**F**), the Cd concentrations in HC1 (**G**) and cell wall (**H**) of roots, and the Fe concentration in HC1 (**I**) and cell wall (**J**) of roots. Different letters above each bar indicate significant difference at *p* < 0.05.

In this study, the localization of NO in Arabidopsis roots was detected by 3-amino,4-aminomethyl-2′,7′-difluorescein, diacetate (DAF-FM DA) staining (Figure 10). Under Cd stress, the SAY09-exposed roots displayed stronger NO-associated fluorescence than the controls. However, upon exposure to NPA treatment, the intensity of NO-associated fluorescence was observably weakened in the SAY09-exposed roots under Cd stress. Furthermore, treatment with 1 mM c-PITO, an NO scavenger, could not mitigate leaf chlorosis in the SAY09-exposed plants (Figure 11A). However, treatment with 50 μM GSNO (NO donor) and SAY09 exposure obviously mitigated the chlorotic symptoms in the Cd-treated plants, which was in accordance with the increment of total chlorophyll content (Figure 11B). Additionally, the GSNO- and SAY09-exposed shoots accumulated more Fe and less Cd levels than the controls. Conversely, more Cd and less Fe accumulation were found in the SAY09-exposed roots compared with the 2-(4-carboxyphenyl)-4,4,5,5-tetramethylimidazoline-1-oxyl-3-oxide (c-PTIO)-treated plants (Figure 11C–E). 

Furthermore, the HC1 content was remarkably higher in both the GSNO- and SAY09-exposed roots under Cd stress than the controls. When plants were treated with c-PTIO, the HC1 content was evidently decreased in the SAY09-exposed roots (Figure 11F). Accordingly, less Cd retention was observed in HC1 and the cell wall of the SAY09-exposed plants upon exposure to c-PTIO treatment (Figure 11G,H). Additionally, the SAY09-exposed plants treated with c-PTIO displayed much higher Fe concentration in HC1 and the root cell wall than plants grown under +SAY09 and +IAA treatments, respectively, under Cd stress (Figure 11I,J).

**Figure 12:**
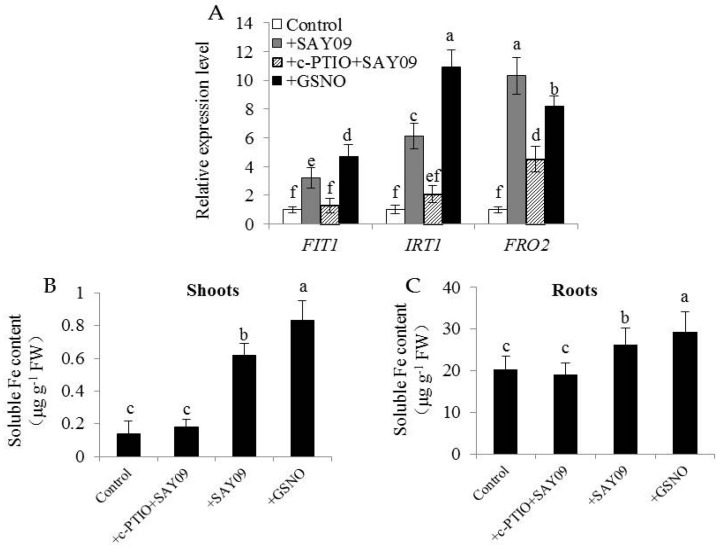
Effects of c-PTIO treatment on the expression of Fe uptake-related genes and soluble Fe content in the SAY09-exposed *Arabidopsis* plants. Six-day-old seedlings were grown on 1/2 MS agar medium with 50 μM CdCl_2_ after 12 d of exposure to 1 mM c-PTIO pulse SAY09 (+c-PTIO+SAY09), SAY09 (+SAY09) or 50 μM S-nitrosoglutathione (GSNO) (+GSNO) treatments, and plants treated with 50 μM CdCl_2_ alone as the controls (Control). Then, these plants were used to analyze the expression of Fe uptake-related genes including *FIT1*, *IRT1* and *FRO2* (**A**), soluble Fe content in shoots (**B**) and roots (**C**). Different letters above each bar indicate significant difference at *p* < 0.05.

The expression levels of *FIT1*, *IRT1* and *FRO2* in plants were further examined. Both GSNO and SAY09 exposure significantly increased the transcription of *FIT1*, *IRT1* and *FRO2* in the Cd-treated plants, and their expression levels were largely inhibited in the SAY09-exposed plants by c-PTIO treatment (Figure 12A). Concomitantly, soluble Fe content of shoots was markedly higher in the GSNO- and SAY09-exposed plants under Cd stress than the control and c-PTIO-treated SAY09-exposed plants (Figure 12B). Similar results were also observed for roots (Figure 12C). Collectively, these findings implied that the SAY09-induced increase of soluble Fe might contribute to promoting the mobilization of cell wall Fe, thereby enabling more polysaccharide (such as HC1) to bind free Cd. 

## 4. Discussion

Complex volatile blends are released by microbes to attract, communicate and defend within their ecological niche [40]. In nature, soil-borne bacteria can emit diverse kinds of VOCs for overcoming physical constraints of soil matters to reach plant roots, even aboveground tissues of plants, thereby affecting various physiological processes in plants [21]. It has recently been shown that the production of VOCs by PGPR strains can regulate Fe and selenium (Se) absorption, photosynthesis, and pathogen invasion [31,32,33,34]. However, whether microbial VOCs induce Cd tolerance of pants and the underlying mechanisms are largely unknown. Here, we reported for the first time that PGPR-emitted VOCs obviously alleviated leaf chlorosis in *Arabidopsis* plants under Cd stress. RNA-Seq analyses revealed that some auxin biosynthetic genes were transcriptionally activated in the SAY09-exposed plants. At the physiological levels, the increased auxin biosynthesis along with enhanced Fe acquisition and reduced shoot Cd content were observed. The SAY09-exposed plants also accumulated more Cd in the root cell wall, whereas this was compromised in plants treated with NPA or c-PTIO. 

In Cd-contaminated soils, plants often exhibit growth tardy, chlorophyll and biomass loss [2]. Cd toxicity is mainly attributable to dysfunction of nutrient element uptake and homeostasis, particularly for Fe nutritional status since the Cd-induced chlorosis and molecular responses are much similar to that imposed by Fe deficiency [6,8]. In this study, the *Arabidopsis* plants displayed chlorotic symptoms with biomass loss, chlorophyll reduction, and excessive ROS accumulation under Cd stress. Moreover, Cd stress markedly decreased shoot and root Fe concentrations, which was in good agreement with the earlier results described by Wu et al. [7]. Importantly, recent studies have indicated that the enhanced Fe acquisition is conducive to improving Cd tolerance in plants [50,51]. Co-overexpression of *FIT* with *bHLH38* or *bHLH39*, belonging to the *Arabidopsis* basic helix-loop-helix (bHLH) transcription factor family, promotes Fe translocation from roots to shoots and augments root Cd sequestration, thereby enhancing Cd tolerance in *Arabidopsis* plants [7]. Transgenic tobacco plants overexpressing the iron transporter *NtPIC1* display more shoot Fe content and lower Cd accumulation compared with the wild-type plants [52]. Moreover, the increased Fe source has been shown to improve Cd tolerance in plants by inhibiting Cd uptake [53]. Here, SAY09 exposure alleviated leaf chlorosis, and led to higher shoot and root Fe content compared with the controls. Concomitantly, shoot Cd concentration was significantly lower in the SAY09-exposed plants than the controls. Thus, the enhanced Fe uptake and its translocation to shoots by SAY09 may confer the strong ability of plants to detoxify Cd. 

Recent studies have shown that Cd exposure induces overproduction of cellular ROS, which triggers oxidative damages to plant cells [54,55]. The MDA and EL levels are recently taken as important indicators of the degree of oxidative damage [25]. In this study, SAY09 exposure significantly decreased the levels of ROS, MDA and IL in the Cd-treated *Arabidopsis* plants compared the controls, implying that the SAY09-exposed plants experienced less oxidative damages imposed by Cd treatment compared with the controls. Interestingly, Cd stress has been shown to enhance the activity of GH3 encoding the auxin conjugate enzyme, thereby decreasing the auxin levels in poplar [56]. In *Arabidopsis* plants, Cd treatment greatly increases the activity of IAA oxidase, leading to reduction of IAA content [18,57]. Similarly, our results revealed that Cd treatment evidently reduced endogenous auxin content in *Arabidopsis* plants. However, the SAY09-exposed plants exhibited the increased auxin content under Cd stress compared with the controls. Recent studies have indicated that the increased auxin levels notably enhanced the ability of plants to detoxify Cd [58,59]. Exogenous IAA ameliorates Cd-induced oxidative damage in wheat by enhancing antioxidant defense activities [60]. Treatment with IAA alleviates Cd toxicity in *Trigonella foenum-graecum* L. plants by regulating the ascorbate–glutathione cycle [59]. Moreover, the auxin-induced mitigation of Cd toxicity in *Arabidopsis* plants is associated with the increased Cd binding capacity of root cell walls [18]. 

To further examine whether the SAY09-induced auxin was involved in activation of the adaptive mechanisms in plants, exogenous NPA was used to treat the SAY09-exposed plants grown under Cd stress. The results revealed that treatment with NPA abrogated the effects of SAY09 on alleviating Cd toxicity in plants. Shoot Cd concentration was significantly increased in the SAY09-exposed plants after NPA treatment. However, the SAY09-exposed roots accumulated lower Cd concentration, implying that auxin positively mediated the accumulation of Cd in roots. It has recently been indicated that the cell wall is the major sites for Cd retention in plants [17,18]. Here, there was more Cd accumulation in the root cell wall of IAA- and SAY09-exposed plants, although the Cd levels were markedly decreased in the SAY09-exposed plants treated with NPA. These findings implied that the auxin-induced Cd accumulation fortified Cd fixation in *Arabidopsis* roots. It is well known that the cell wall primarily constitutes cellulose and matrix polysaccharides such as pectin and hemicellulose [18]. The cellulose is generally considered as chemically inactive molecules, and the HC1 of root cell wall possesses the strong ability to bind metal ions in *Arabidopsis* plants [17]. In this study, either IAA or SAY09 exposure considerably augmented both the HC1 content and Cd levels in HC1 of root cell wall under Cd stress compared with the controls. In contrast, SAY09 exposure had almost no impacts on pectin content and Cd retained in pectin. 

Besides auxin, some other hormones such as ABA and GA have also been shown to alleviate Cd toxicity in plants [12,13]. During Cd exposure, treatment with ABA results in lower Cd content in rice (*Oryza sativa* L.) plants than the controls [60]. Exogenous treatment with ABA confers increased Cd tolerance in rice plants by decreasing Cd translocation from roots to shoots [61]. More recently, ABA exposure markedly lessens Cd accumulation in *Arabidopsis* plants by inhibiting the expression of Cd transporter gene *IRT1* [13]. Similarly, the GA-alleviated Cd toxicity is positively mediated by reducing the transcription of *IRT1* in *Arabidopsis* plants [13]. In the present study, the SAY09-exposed plants displayed lower ABA accumulation in plants under Cd stress compared with the controls. RNA-Seq analyses further revealed that SAY09 exposure significantly suppressed the ABA-related signaling pathways in the Cd-treated plants. Additionally, the SAY09-exposed plants did not exhibit higher GA levels under Cd stress than the controls. Hence, the enhanced Cd tolerance of plants by SAY09 was not mainly attributable to induction of ABA and GA biosynthesis. 

To survive in Cd-contaminated soils, plants have to overcome some major challenges such as Fe absorption and Cd toxicity [7]. Cd stress has been shown to inhibit Fe absorption and stimulate Fe deficiency responses, thereby causing Fe deficiency-induced leaf chlorosis in plants [6,7,8]. Intriguingly, the enhanced Fe acquisition or increased Fe source can improve Cd tolerance in plants [7]. NO is an important signal molecule that plays essential roles in the regulation of Fe deficiency responses in plants [50,62,63,64,65]. In *Arabidopsis*, Fe deprivation-induced increase of NO contributes to improving Fe availability by mediating the expression of Fe uptake-related genes [49,62]. Accordantly, Fe deficiency triggered the NO accumulation in the woody plants, which is required for activating a series of adaptive responses to Fe deficiency [65]. Interestingly, Fe can also react with NO to form nitrosyl-Fe complexes, which increase the availability of metabolically active Fe within the plant [63]. Furthermore, NO act as downstream signals of auxin to regulate the transcription of Fe uptake-related genes in Fe-deficient plants [50]. Here, the SAY09-exposed plants displayed a significant increase of auxin and NO accumulation in the Cd-treated plants. However, treatment with NPA could not alleviate Cd toxicity and greatly inhibited the auxin-induced NO synthesis in the Cd-treated plants. Hence, the SAY09-induced auxin likely promoted the NO accumulation, thereby activating a series of adaptive mechanisms in plants under Cd stress. Similar results were observed in some recent reports, calcium chloride mediates the alleviation of Cd toxicity in rice by elevating NO levels [14]. Alleviated Cd toxicity in wheat plants by ascorbic acid is primarily mediated by NO signaling pathways [33]. Thus, keeping higher NO level was beneficial to increase Cd resistance of plants. 

To confirm this assumption, we examined the effects of c-PTIO on the SAY09-exposed plants grown under Cd stress. As expectedly, SAY09 exposure could not mitigate Cd toxicity in the c-PTIO-treated plants, while exogenous GSNO remarkably increased Cd resistance in plants. Both SAY09 and GSNO exposure notably upregulated the expression of Fe uptake-related genes and further augmented Fe uptake in plants under Cd stress. We also found that cell wall-retained Fe was observably lower in both the SAY09- and GSNO-exposed plants under Cd stress compared with the controls. Consistently, the SAY09- and GSNO-exposed plants displayed higher soluble Fe content than the controls. However, treatment with c-PTIO largely blocked the SAY09-mediated Fe uptake and reutilization of cell wall Fe in the Cd-treated plants. It has previously been shown that exogenous GSNO promotes the biosynthesis of HC1 and increases the ability of cell wall to bind Cd in plants, contributing to alleviation of Cd toxicity [17]. Here, SAY09 exposure increased HC1 content and Cd fixation in root cell wall in plants, whereas these effects were greatly abolished in the c-PTIO-treated plants. Thus, SAY09 exposure improved Fe acquisition and Cd fixation in the root cell wall, which primarily contributed to activation of NO-mediated signaling pathways. 

## 5. Conclusions 

**Figure 13:**
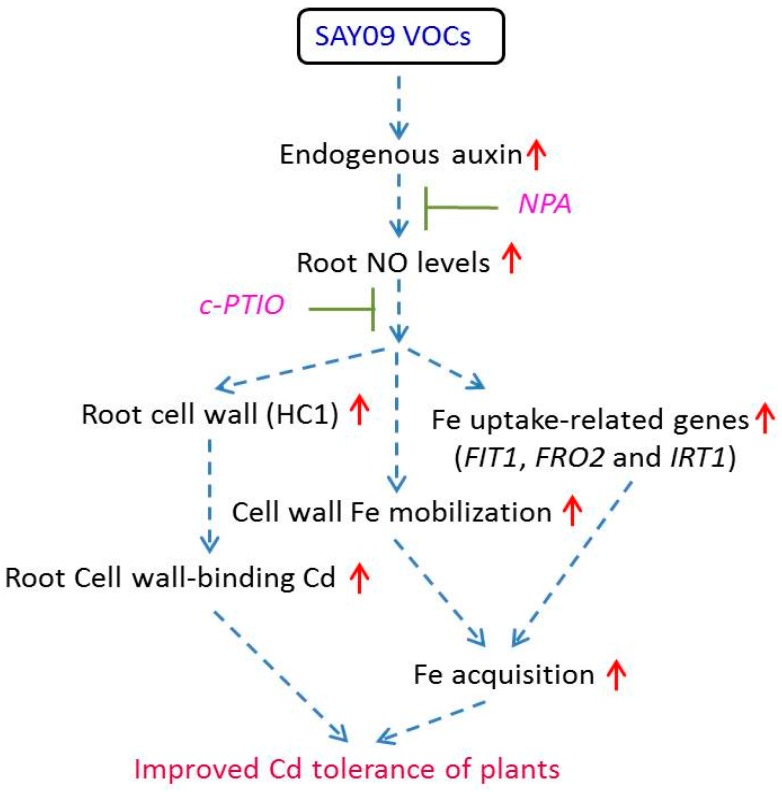
A schematic model of NO signals acting downstream of SAY09-induced auxin to enhance Cd tolerance in *Arabidopsis* plants. SAY09 exposure could induce a great increase of auxin levels in roots with the subsequent stimulation of NO synthesis. The enhanced NO signals then activate diverse adaptive mechanisms such as enhanced cell wall-retained Cd and Fe acquisition, which confers increased Cd resistance of plants. Dashed arrows denote regulatory pathways. Red upright arrows denote a significant increase in content or effects.

A model was proposed linking the SAY09-induced increase of auxin levels to plant’s adaption to Cd stress (Figure 13). The results indicated that SAY09 exposure led to a significant increase of endogenous auxin in *Arabidopsis* plants under Cd stress, and further activated a series of adaptive mechanisms such as increased Cd fixation in the root cell wall and Fe uptake-related genes, which were closely associated with the actions of NO. Moreover, SAY09 exposure promotes the remobilization of cell wall Fe and increased soluble Fe levels under Cd stress, thereby allowing more cell wall polysaccharide to bind Cd. Collectively, the collaborative effects contributed to ameliorating Cd-induced leaf chlorosis in plants.

## Supplementary Materials

The following are available online at www.mdpi.com/2073-4425/8/7/173/s1. Table S1: Primers used in this study. Table S2: Volatile organic compounds produced by *Bacillus amyloliquefaciens* SAY09 in MS media after 12 d of growth and identified by solid-phase microextraction and gas chromatography (GC)–mass spectrometry (MS). Table S3: Statistics of RNA-Seq and mapping. Table S4: Upregulated and downregulated genes differentially expressed in the Cd-treated *Arabidopsis* plants compared with non-treated plants. Table S5: Upregulated and downregulated genes differentially expressed in the SAY09-exposed plants grown under Cd stress compared with the control (Cd-treated) plants. Table S6: Differential expression of some auxin biosynthetic genes in both the control and SAY09-exposed plants grown under Cd stress. Figure S1: Measurement of the Cd content in the aboveground tissues of *Arabidopsis* plants grown under Cd stress. Figure S2: List of the predominant GO terms for downregulated differentially expressed genes (DEGs) based on GO classifications. GO terms were categorized into three groups: biological process, cellular component and molecular function. (A) NT (non-treated plants) and versus CT (Cd-treated plants). (B) CT (Cd-treated plants) versus CS (Cd-treated SAY09-exposed plants). *p*-value indicates significant difference among NT, CT and CS libraries. Figure S3: qRT-PCR analyses of randomly selective genes including *TCP1*, *NIT1*, *XTH12*, *CYP81D1*, *PDF2.1*, *PUP4*, *PRP1* and *LTP2* among the DEGs.
