# Supplementary figures and images for: Bacillus amyloliquefaciens SAY09 Increases Cadmium Resistance in Plants by Activation of Auxin-Mediated Signaling Pathways
Source: Genes (Basel). 2017 Jun 28;8(7):173. doi: 10.3390/genes8070173 (PMC5541306; doi:10.3390/genes8070173)

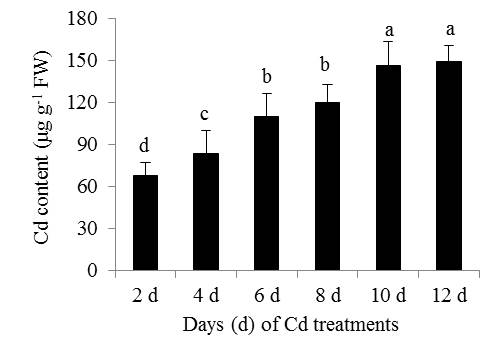

Supplement: Supplementary file 1 [file genes-08-00173-s001.zip › Supplementary materials/Supplementary Figure1.jpg]

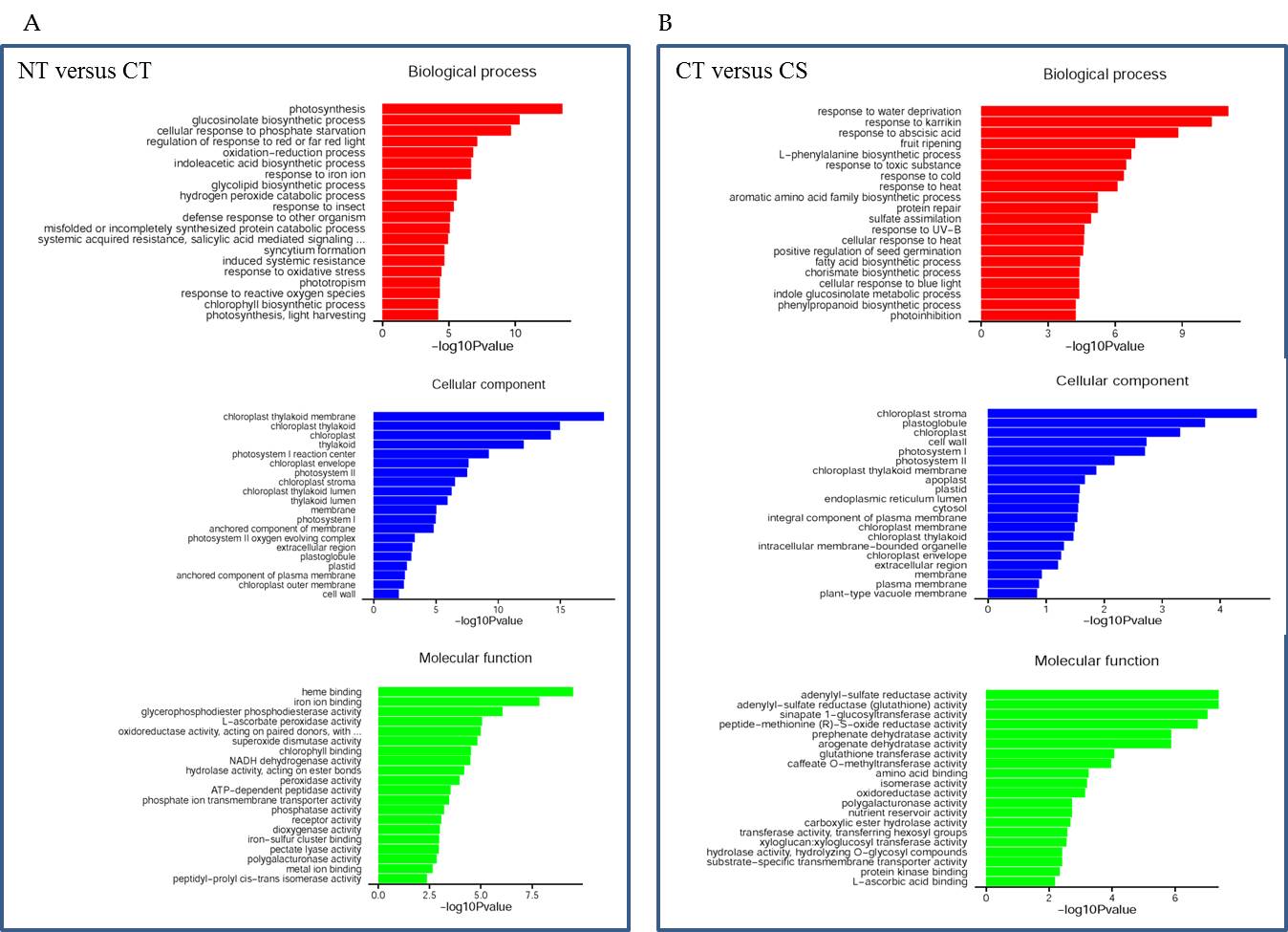

Supplement: Supplementary file 1 [file genes-08-00173-s001.zip › Supplementary materials/Supplementary Figure2.jpg]

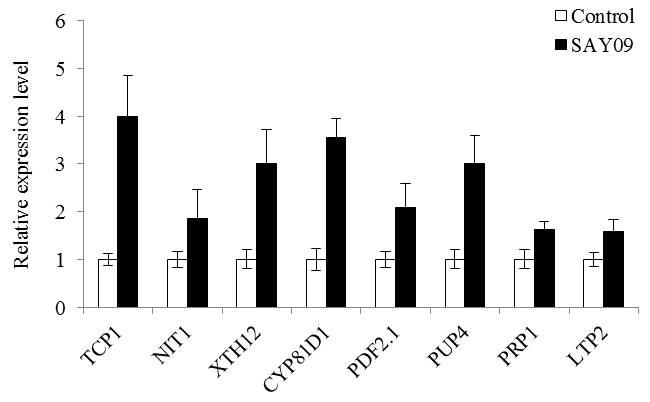

Supplement: Supplementary file 1 [file genes-08-00173-s001.zip › Supplementary materials/Supplementary Figure3.jpg]
